# Ultrasmall CuS@BSA nanoparticles with mild photothermal conversion synergistically induce MSCs-differentiated fibroblast and improve skin regeneration

**DOI:** 10.7150/thno.39471

**Published:** 2020-01-01

**Authors:** Yao Xiao, Jinrong Peng, Qingya Liu, Lijuan Chen, Kun Shi, Ruxia Han, Qian Yang, Lin Zhong, Ruoyu Zha, Ying Qu, Zhiyong Qian

**Affiliations:** 1 State Key Laboratory of Biotherapy and Cancer Center, West China Hospital, West China Medical School, Sichuan University and Collaborative Innovation Center of Biotherapy, Chengdu 610041, China; 2 The Department of Radiology, Henan Key Laboratory of Neurological Imaging, Henan Provincial People's Hospital & the People's Hospital of Zhengzhou University, Zhengzhou 450003, China; 3 The School of Pharmacy, Chengdu Medical College, Chengdu, 610500, China

**Keywords:** MSCs, differentiation, CuS@BSA, photothermal conversion, wound healing.

## Abstract

Mesenchymal stem cell (MSC)-based therapies have been used in skin regeneration due to their ability to differentiate into many cells, promote cytokine secretion and participate in collagen deposition. In this study, we concluded that a CuS@BSA nanoparticles exhibited similar potential in inducing MSCs differentiation to fibroblasts as Cu ions for wound healing.

**Methods**: First, we verified the photothermal efficiency of CuS@BSA *in vivo* and *vitro* and had no cytotoxicity for MSCs when the temperature was controlled at 42 °C by adjusting the power of irradiation at 980 nm. And then we detected the expression of vimentin in MSCs, which further directed the MSCs to fibroblasts through Western blotting and Immunofluorescence when treated with CuS@BSA or pre-heat at 42 °C. In addition, we implanted MSCs into the Matrigel or electrospun PLA nanofiber membrane *in vitro* to evaluating the effect of heating or CuS@BSA on the morphological change of MSCs by SEM. Finally, we evaluated improving skin regeneration by the combination of preheated-MSCs and CuS@BSA nanoparticles that were encapsulated in Matrigel.

**Results**: The CuS@BSA nanoparticles have good photothermal conversion efficiency. Not only CuS nanoparticles itself or after irradiation at 980 nm stimulated the expressioin of vimentin in MSCs. Besides, the CuS@BSA can promote cell proliferation as Cu ion through the expression of ERK. The combination of the CuS@BSA nanoparticles and thermal treatment synergistically improved the closure of an injured wound in an injured wound model.

**Conclusions**: MSCs combined with CuS@BSA are a promising wound dressing for the reconstruction of full-thickness skin injuries.

## Introduction

Mesenchymal stem cells (MSCs) are multipotent stem cells with commonalities of stem cells, namely, self-renewal and multidirectional differentiation 1, 2. MSCs are present not only in the bone marrow but also in the skeletal muscle, epithelium, and trabecular bone. MSCs have great value in clinical applications because of their strong proliferative capacity, potential in multidirectional differentiation, immunomodulatory properties 3, 4, and ease of isolation and expansion. MSCs *in vivo* can be activated and mobilized if need. For example, MSCs have been used in the therapy of some autoimmune diseases, multiple sclerosis, systemic lupus erythematosus, and systemic sclerosis 5, 6. In addition, they involve the regeneration of bone, cartilage, and joints and the repairing of spinal cord injuries and nervous system diseases, though the efficiency is low. The further study of the mechanisms of MSC behaviors may provide avenues for increasing their capacity for tissue repair 7, 8.

Bone marrow-derived mesenchymal stromal cells (BM-MSCs), as a source of MSCs, have been widely used in tissue repair, for bone as well as skin 9, 10. BM-MSCs have been applied to different dermal matrices in small 11, 12 and large 13 animal models with beneficial effects on vascularization and wound healing. Fierro et al. implanted BM-MSCs in a three dimensional scaffold for dermal regeneration (SDR), resulting in promoted endothelial cell migration and accelerated wound healing by hypoxic preconditioning of seeded dermal scaffolds 14. Endothelial cells differentiating from BM-MSCs can be directly integrated into newly developing microvascular networks during wound healing 11, 15. Recently, MSC-based therapies for burn healing and re-epithelization of chronic ulcerated skin have made significant progress 16, 17.

Wound healing is a complex and interactive process that involves acute inflammation, re-epithelialization, angiogenesis, granulation tissue, and tissue remodeling. Healing requires interactions between cells, extracellular matrix (ECM), and other components 18. When trauma occurs, the defect is quickly covered by a mixture of cytokines released from the mesothelial cells, fibrin, and coagulated blood. The wound is then stabilized by cross-linking the fibrin, collagen, and other matrix components mediated by fibronectin. And then granulocytes, monocytes, and macrophages are recruited into the wound area and the fibrin clot. Additionally, macrophages and granulocytes also infiltrate the fibrin clot to prepare for the regeneration of fibroblastic organized fibrin bands and permanent adhesion, which is critical in the reconstruction of blood vessels and nerve fibers. Angiogenesis and the migration of basal epithelial cells into the boundary between the blood clot on the surface and the granulation tissue occur. All these processes require different cell types and their related phenotypes, especially the fibroblast which plays a critical role in skin regeneration. Cell-based skin tissue regeneration can be achieved by MSC-induced vascular endothelial growth factor (VEGF) production as well as by the participation of MSCs in collagen deposition for dermal regeneration 19.

Various agents, including copper, have been used to induce MSC differentiation into expected phenotypes. Copper is an indispensable trace element in living organisms and is often used as an enzyme cofactor to drive important physiological processes including cellular respiration, neurotransmitter transmission, iron ion uptake and anti-oxidative stress 20. Turski, M. L. et al. illustrated that copper plays a well-established structural role in proteins, including in metalloregulatory transcription factors in fungal and in copper transporter receptor1(Ctrl1), which mediates the phosphorylation of ERK1/2 to promote cell proliferation and migration especially in tumorigenesis^21^-^23^. Christopher M. Counter et al. suggested that combining a Cu chelator and MERK inhibitor may merit clinical consideration for the treatment of BRAF mutation-positive cancer and cancers developing resistance to MEK1/2 inhibitors 24, further demonstrating the potential of Cu in inducing cell differentiation.

Furthermore, the addition of Cu can enhance angiogenesis by stabilizing the expression of hypoxia-inducible factor (HIF-1α) and activate ERK, which may both favor the acceleration of wound healing 25. A porous Cu-BG/ESM nanocomposite film for wound healing of skin tissue was prepared because of the improvement of angiogenesis by copper ions via the stabilization of the expression of HIF-1α and secretion of VEGF 26, 27. However, the elevated nonphysiological concentrations of copper ions can be toxic because the ions can interfere with the homeostasis of other metals, damage DNA, and generate reactive oxygen species that can adversely modify proteins, lipids, and nucleic acids 28, 29. Although a slowly releasing copper system may help to reduce the cytotoxicity of copper *in vitro* and enhance wound closure rates *in vivo* 30, lower toxicity candidate is still needed.

Nanoparticle-based strategies may provide an alternative. In recent years, the nanobiological effects of nanobiomaterials have been identified. The functions of some nanoparticles can even act as a substitution in mediating the cell behaviors, e.g., the Fenton reaction mediated by iron oxide nanoparticles 31. In addition, Li Mu et al. used a hybrid hydrogel system with CuS NPs, which the copper ions can stimulate fibroblast proliferation and angiogenesis effectively, and the photothermal and photodynamic properties of CuS under 808 nm NIR light irradiation can inhibit the viability of *Staphylococcus aureus* and* Escherichia coli up* to 99%^32^, ^33^. Some other melt irons like Au, Ag. Ti which has the photothermal property can also act as an antibacterial agent and promote fibroblast proliferation and differentiation though the photothermal property. Furthermore, Cu-based chalcogenides, as well as CuS, have been widely proven as effective photothermal agents (PTAs) due to their intense near infrared (NIR) absorption. A copper silicate hollow microsphere (CSO HMS)-incorporated electrospun scaffold has been evaluated in the application of melanoma therapy and demonstrated to further improve the regeneration of skin tissue after photothermal therapy (PTT). Cu ions in this hybrid system promoted cell migration, angiogenesis, and collagen deposition 34, indicating the potential of Cu-based nanomaterials in mediating cell differentiation.

Therefore, in this study, we plan to further study the influence of ultrasmall CuS@BSA nanoparticles onto the differentiation of MSCs, and their improvement in skin regeneration. Additionally, given CuS@BSA is an excellent agent for photothermal therapy of cancer, the effect of heating (especially mild heating) onto the differentiation of MSCs remains unclear. Herein, we evaluate the effect of heating on the differentiation of MSCs and improvements in skin wound regeneration. CuS@BSA was prepared as previously reported 30, 35, 36. Cultrex^®^ Reduced Growth Factor Basement Membrane Extract (referred to as Matrigel) was introduced to provide appropriate cellular attachment and new matrix formation with a low growth factor. For comprehensive understanding of the mechanism of how Cu induces MSC differentiation into fibroblasts *in vivo* and *in vitro*, an investigation of the properties, characteristics, cell bioactivity, and full thickness wound healing of CuS@BSA combined with MSCs dressing was conducted in detail.

## Materials and methods

### Materials and animals

CuS@BSA and PLA electrospinning film was previously synthesized by our group. Primary antibody vimentin (#5741), β-actin (#3700), ERP/P-ERK (#9926), second antibody conjugated Alexa Fluor^®^ 488(#4408) and Alexa Fluor^®^ 647 (#4418) were purchased from Cell signaling technology company. The second antibody conjugated HRP (goat-anti-mouse and goat-anti-rabbit) were purchased from Proteintech. Cultrex^®^ Reduced Growth Factor Basement Membrane Extract (#3533-005-02) was purchased from Trevigen.

Tegaderm film was purchased from 3M Health Care. Puromycin (A1113803) was purchased from Thermo Fisher Scientific. D-Luciferin sodium salt (308290) was purchased from J&K Scientific.

Male Sprague-Dawley (SD) albino rats and BALB/c mice were purchased from the Laboratory Animal Center of Sichuan University. All the rats were housed individually to prevent fighting and attack to the wounds, and received food and water *ad libitum*. All the animals were quarantined for a week before treatment. All animal procedures were performed following the protocol approved by the Institutional Animal Care and Treatment Committee of Sichuan University (Chengdu, P.R. China). All animals were treated humanely throughout the experimental period.

### Perparation of CuS@BSA

CuS@BSA NPs were prepared according to a biomineralization strategy in aqueous solution at physiological temperature (37 °C). Typically, an aqueous CuCl_2_•2H_2_O solution (0.05 mmol, 5 mL, 37 °C) and a BSA solution (50 mg/mL, 5 mL, 37 °C) were mixed in a one-necked flask (25 mL) with magnetic stirring in a water bath (37 °C). Upon mixing, a light green turbidity appeared. Subsequently (3 min later), a NaOH solution (1 M, 500 µL) was introduced to adjust the pH of the system to ~12, and the mixture became a transparent deep blue. Subsequently, 400 µL of Na_2_S•9H_2_O (242.16 mg/mL) was quickly injected into the above system, and the solution turned to a deep brown. After 4 h, the reaction was completed, and the solution was dialyzed (MWCO: 8000~14000 Da) against deionized water for 48 h to remove excess Cu^2+^ and alkaline solution. Upon lyophilization, a dark green cotton-like powder was collected and redissolved in 3 mL of PBS (1×, pH = 7.4) for further use. The exact concentrations of Cu^2+^ were measured using ICP-AES.

### Characterization of CuS@BSA *In vitro*

Particle size distribution of prepared CuS@BSA was determined by dynamic light scattering (DLS) using a Malvern Nano ZS90 laser particle size analyzer at 25°C. All results were the mean of the three different samples and were expressed as the mean±SD.

The morphological characteristics of CuS@BSA were examined using a transmission electron microscope (TEM, H-6009IV, Hitachi, Japan). CuS@BSA was diluted with distilled water and placed on a copper grid covered with nitrocellulose.

CuS@BSA was irradiated from the right side with a 980-nm laser at a power density of 2 W/cm^2^ for 5 min, and the temperature was recorded by a Fluke TI32 infrared (IR) thermal camera (Infrared Cameras, Fluke, Avery, WA, USA) until the sample reached room temperature.

A full wavelength scan (200-1100 nm) of the absorption spectrum was performed in an aqueous solution of 3 mL CuS@BSA in a UV cuvette.

The photothermal conversion efficiency of CuS@BSA was calculated as follow according to Zhang shaobo et al^37^. The photothermal conversion efficiency (ƞ) was calculated according to Equation (1):

η 
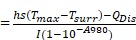
 (1)

Where, 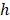 represents the heat transfer coefficient, 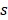 is the surface area of the container, 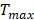 represents the maximum steady-state temperature (51.2 °C), 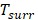
is the ambient temperature of the environment (23.5 °C), 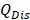 represents the heat dissipation from the light absorbed by the solvent and the quartz sample cell (0.225 W), 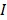 is the incident laser power (1.42 W/cm^2^ ), and A980 is the absorbance of CuS@BSA (100 μg/ml) at 980 nm (0.177). The value of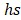 is derived from Equation (2):


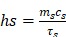
 (2)

Where 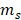 and 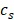 are respectively the mass (1.6154 mg) and heat capacity (4.2 J/g) of the deionized water used to disperse the CuS@BSA. 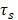
is the time constant for heat transfer of the system. The value of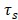 is calculated according to Equation (3) (4):


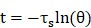
 (3)


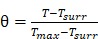
 (4)

From Figure S1B, 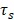
was calculated to be 447.93, According to the obtained data and Equation (1), the photothermal conversion efficiency of the CuS@BSA was determined to be 42%.

### *In vitro* Biocompatibility and Cell Differentiation

#### Cells and Cell Culture

Mesenchymal stem cells (MSCs) were isolated from newborn SD/BALB/c mice bone marrow and cultured in Dulbecco's modified Eagle medium with 1 mg/ml glucose (DMEM, Invitrogen, CA) supplemented with 10% fetal bovine serum(FBS, Gibco, US). The mononuclear cell (MNC) fraction of bone marrow aspirates was obtained by density gradient centrifugation [Percoll (1.073 g/l) for 30 min at 700 × g] and plated in plastic culture flasks with MSCs culture media. After 3 days, non-adherent cells were removed by 2-3 washing steps with PBS and refreshed by DMEM medium. All the cells were incubated at 37 °C in a humidified atmosphere with 5% carbon dioxide.

#### Cells Infection

Lentivirus carrying pLenti-CMV-EGFP-linker-Luc-PGK-Puro was purchased from OBio Technology Company to construct MSC cells stable expressed luciferase.

Cells were seed in a 24-well plate at approximately 1×10^5^ cells for 24 h, then add 250 μl of prewarmed medium without FBS and Lentivirus was added, and the medium refreshed with 10% FBS after 12-24 h. The expression of luciferase used D-Luciferin sodium salt by multifunction microplate reader.

#### Cell Differentiation and Proliferation

MSCs cultured in PLA electrospinning film or Matrigel in a transwell were treated with CuS@BSA and then fixed with 4% paraformaldehyde (w/v) for 30 min, and gradient dehydrated by 50%, 60%, 70%, 80%, 90%, and 100% ethanol. The samples were freeze-dried and analyzed by SEM (JSM-7500F, JEOL, Japan).

The uptake of CuS@BSA and CuCl_2_ was performed as follow: MSCs were seeded in 3-cm dish (50000 cells) overnight and treated with CuS@BSA (50 μg/ml) or CuCl_2_ (5 μg/ml). After 24h, 48h, 72h, cells were collected and digested by the mixture of hydrochloric acid and nitric acid, and then detected using ICP-AES.

Cell viability was determined by the MTT assay (Roche Diagnosis, Indianapolis, IN). Briefly, the MSCs seeded in 96-well plates (5000 cells/well) were treated with CuCl_2_ and CuS@BSA at indicated doses. 24 hours after treatment, the cell viability was determined using a UV spectrophotometer at 570 nm.

MSCs were seeded in cell culture E-plates at a cell density of 5000 cells/well and incubated overnight in culture medium at 37 °C and 5% CO_2_. Cells were then starved with medium containing CuS@BSA and CuCl_2_ in a different dose. The cell growth curves were automatically recorded on the xCELLigence System (Roche Applied Sciences) in real-time. The cell index was followed for 3 days.

#### Expression of the Fibroblast-Related Genes

The related gene of fibroblasts vimentin was detected by western blotting, qPCR, immunofluorescence and confocal microscopy.

The whole proteins were extracted from MSCs treated with CuCl_2_ or CuS@BSA by Mammalian Protein Extraction Reagent (CW BIO in China) and tested according to the protocols with the antibody against vimentin, ERK/p-ERK, and β-actin.

The total mRNA was extracted from MSCs treated with CuCl_2_ or CuS@BSA by Trizol Reagent (TaKaRa Bio.) according to the manufacturer's instructions. The PCR primers corresponding to vimentin and β-actin functional gene sequences were synthesized by TaKaRa, and the sequences were as following: for vimentin (sense: 5'-AACCTGGCCGAGGACATCAT-3'; antisense: 5'-CCTGCAAGGATTCCACTTTACG-3'; for β-actin (sense: 5'-ACGGTCAGGTCATCACTATCG-3'; 5'-GGCATAGAGGTCTTTACGGATG-3'). The cDNA was synthesized by the ReverTra Ace^®^qPCR RT Master Mix with gDNA Remover (TOYOBO Bio) according to the manufacturer's instructions. And the qPCR were followed by 40 PCR cycles, each with temperature variations as follows: 98 ^o^C for 10 s, 60 ^o^C for 30 s. The PCR results were shown as ΔCt of vimentin to β-actin.

The cells were seeded onto coverslips in a 6-well plate 48 h after treatment and fixed with 4% paraformaldehyde (w/v) for 30 min, and they were washed for 10 min with PBS twice, and permeabilized with 0.2% (w/v) Triton X-100 in PBS for 2-5 min. The blocking step was performed for 30 min in PBS containing 10% bovine serum albumin (BSA). Cells were then incubated for 4 hours with the primary vimentin or/and β-actin antibodies diluted in PBS containing 1% BSA at RT. After being washed with PBS three times, cells were incubated for 1 h with secondary fluorescein isothiocyanate or tetramethyl rhodamine isothiocyanate-conjugated antibodies. After several additional washing steps, the cells were stained with DAPI (Beyotime, China). The expressions of vimentin and β-actin protein were assessed using a Leica DM 14000B confocal microscope.

### *In vivo* Evaluation of Wound Healing

#### Animal Experiments

Twenty adults Sprague-Dawley (SD) rats (2 months old; 220-250 g) and two BALB/c mice were used in this study. BALB/c mice were anesthetized with chloral hydrate sodium (200 mg/kg) via intraperitoneal injection. The wound areas were marked and then sterilized with iodine prior to incision. MSCs-Luc and MSCs-Luc with Matrigel were then placed on a skin defect or subcutaneously injected, respectively.

The SD rats were anesthetized with chloral hydrate sodium (250 mg/kg) via intraperitoneal injection. The wound areas were marked and then sterilized with iodine prior to incision, and randomly divided into different groups (5 rats per group). The materials and cells were placed on each skin defect, and Tegaderm film was placed over the wound to cover and fix the dressing.

### Preheated MSCs and NIR Irradiation Treatment IR Thermal Imaging

MSCs were seeded in a 10-cm dish, and when the cells reached approximately 80-90% confluency, they were treated with a 42 °C water bath for 5 min. The cells were collected, mixed with Matrigel or CuS@BSA and implanted in wound site. Alternatively, cells were mixed with CuS@BSA and Matrigel was implanted in wound site and then irradiated with a 980-nm laser at a different power density of 1 W/cm^2^ for 5 min.

CuS@BSA with Matrigel was implanted subcutaneously into the backs of anesthetized mice and then irradiated with a 980-nm laser at a power density of 1 W/cm^2^ for 5 min. The temperatures of the back tissues were recorded during NIR irradiation.

#### Bioluminescence Image

BALB/c mice were injected intraperitoneally with D-luciferin sodium salt solution (15 mg/mL, 200 μL) and then anesthetized with chloral hydrate sodium (200 mg/kg) via intraperitoneal injection. The bioluminescence was detected (PerkinElmer, IVIS Lumina) within 10 min.

#### Wound Area Measurement

On days 7 and 12 post surgery, the Tegaderm film on the wounds were moved, and each defect was photographed by a digital camera. The size of the wound was measured using Image J and calculated by the following formula:

Wound Closure=(A_0_-A_t_)/A_0_×100

where A_0_ refers to the wound area (t=0), and A_t_ refers to the wound area at the on day 7 and 12.

#### Histological and Immunohistochemical Observation

The wounds were removed along with the surrounding healthy skin after the rats were sacrificed. The skin specimens were fixed in 10% formalin for 2 days, dehydrated with a graded series of ethanol, and embedded in paraffin. The sections were stained with hematoxylin and eosin (H&E) and Masson's trichrome. All sections were analyzed by two pathologists in a blinded manner using light microscopy (Olympus BX 45, Olympus).

#### Statistical Analysis

All statistical analysis was carried out using SPSS 20.0 software (Chicago, IL, USA). The results were reported as the mean SE±SD. Values of P < 0.05 were considered to be statistically significant.

## Results and Discussion

### Characterization, photothermal conversion and cytotoxicity of CuS@BSA *in vitro* and *in vivo*

**Figure 1:**
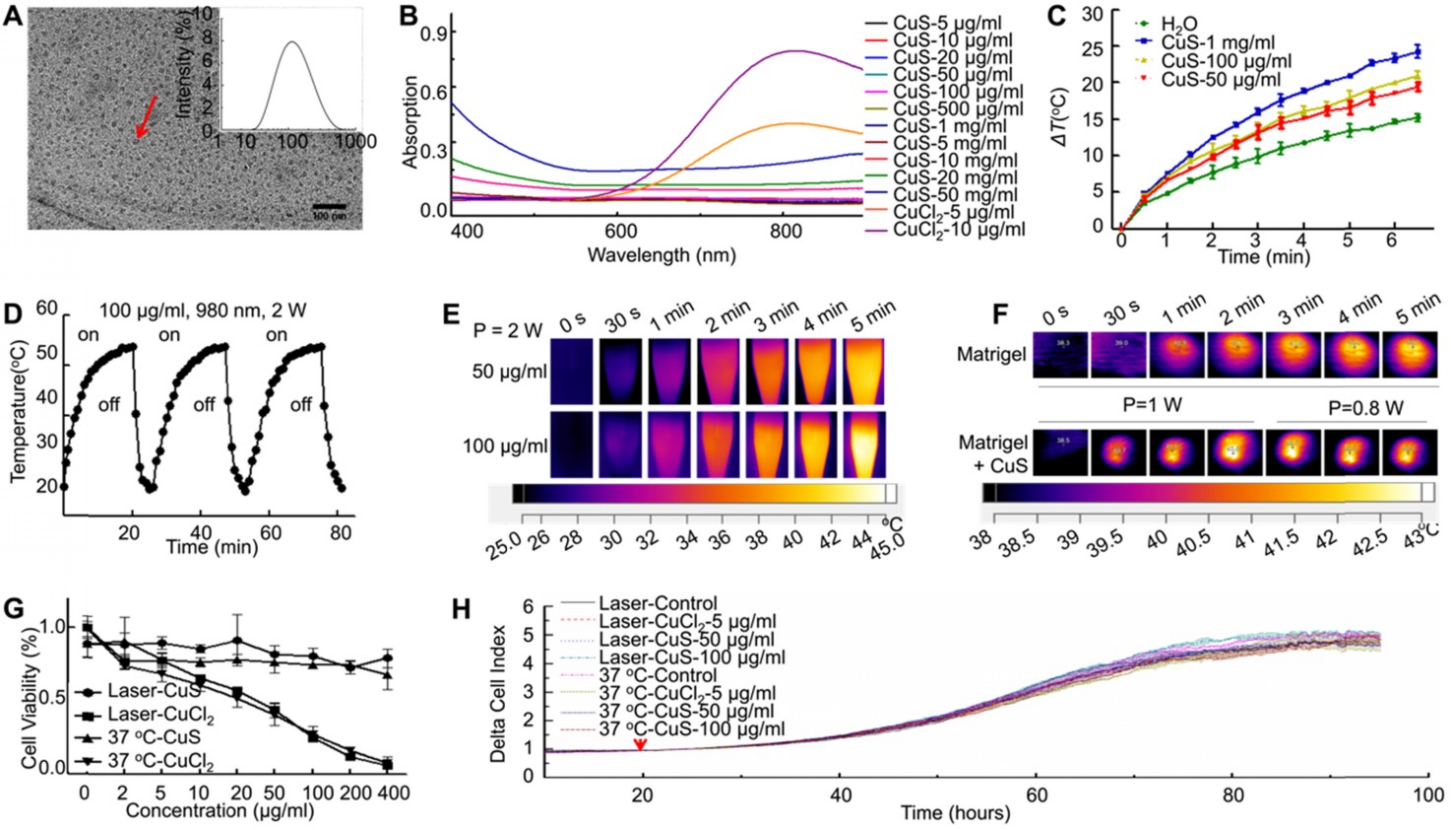
** The characteristics of CuS and cell biocompatibility *in vitro* and *in vivo*.** A) Particle size distribution of CuS@BSA and TEM image; B) UV-vis absorption spectra of CuS@BSA in different doses and CuCl_2_; C) Photothermal conversion of CuS@BSA *in vitro* under irradiation of 980 nm at P=2 W. D) The heat-cool loop of CuS@BSA *in vitro* under irradiation of 980 nm at P=2 W for 3 on and off cycles. E) IR thermal images of CuS@BSA dispersion *in vitro* under irradiation of 980 nm. F) IR thermal images of Matrigel or CuS@BSA with Matrigel dispersion *in vivo* under irradiation of 980 nm. The power transitions from 1 W to 0.8 W in CuS@BSA with Matrigel group to maintain 42 °C; G, H) MSCs survival after coculturing with CuS@BSA and CuCl_2_ with or without laser irradiation by RTCA and MTT.

The CuS@BSA was prepared according to previous reports with some modifications. The average hydrodynamic particle size of the obtained CuS@BSA nanoparticles measured by DLS was 200±2.2 nm, with polydisperse index (PDI) of 0.224±0.015 (Figure 1A insert). However, based on TEM observation, the diameter of CuS@BSA was just several nm (5-8 nm) (Figure 1A), indicating that in aqueous dispersion, the ultrasmall CuS@BSA nanoparticles trend to aggregate to form larger nanoparticles (nanoclusters may be formed) with more stability. We further investigated the optical properties of CuS@BSA in different doses and CuCl_2_ at 5 μg/ml and 10 μg/ml by a UV-visible spectrum (Figure 1B). The results revealed that the CuS@BSA absorbs in the NIR region. Thus, we investigated the photothermal conversion of CuS@BSA in ddH_2_O. When the concentration of CuS@BSA was 50 μg/ml, the temperature of the aqueous dispersion rose from 28.5 to 42 °C in 3 min under irradiation with a 980 nm laser (2 W/cm^2^). As the concentration increased to 100μg/ml and 1 mg/ml, the temperature reached 42 °C and 44.5°C, respectively (Figure 1C). The heat-cool loop of CuS@BSA at 100 μg/ml was performed by irradiation with a 980 nm laser (2 W/cm^2^) under the “on-off” cycles 3 times (Figure 1D). And the photothermal conversion efficiency was calculated to be 42% according to the Equation (1)-(4) (Figure S1A and B). Additionally, the IR thermal imaging visualized the process of heat generation of CuS@BSA in 50 μg/ml and 100 μg/ml (Figure 1E). We further evaluated the photothermal conversion of CuS@BSA *in vivo*. The temperature of CuS@BSA with Matrigel increased from 38.5 °C to 43.8 °C in 2 min under irradiation at 980 nm (1 W/cm^2^), and then the laser power reduced to 0.8 W/cm^2^ to maintain the temperature at 42 °C for low heat stimulation (Figure 1F). The temperature of CuS@BSA with Matrigel dispersion can be maintained at a steady value, and the equilibrium values can be controlled by adjusting the input power of the laser. The results demonstrated that the CuS@BSA nanoparticles were successfully prepared and can be serve as a carrier for photothermal conversion.

Additionally, the obtained CuS@BSA exhibited lower cytotoxicity to MSC cells. From Figure S1C, the uptake of CuS@BSA is higher than CuCl_2_ at 24h, but there was no difference in the uptake of CuS@BSA and CuCl_2_ at 48h and 72h. It reminded us the CuS@BSA was absorbed easier by MSCs than CuCl_2_.Beside several cell death was observed in the MSCs cocultured with CuS@BSA (even the concentration of Cu is as high as 400 µg/mL). Alternatively, dramatic cell death occurred with MSCs cocultured with CuCl_2_, indicating the toxicity of Cu ions (Figure 1G). The introduction of the laser during cell culturing had no influence on the cytotoxicity of Cu-based formulations. Furthermore, we used RTCA to investigate the effect of the introduction of CuS@BSA and laser irradiation on the proliferation of MSCs (Figure H). The results revealed that the addition of CuS@BSA or introduction of the laser irradiation had no significant effect on the proliferation of MSCs (Figure 1 G), further demonstrating the safety and biocompatibility of CuS@BSA compared with CuCl_2_.

### CuS@BSA-induced MSCs differentiation to fibroblast

**Figure 2:**
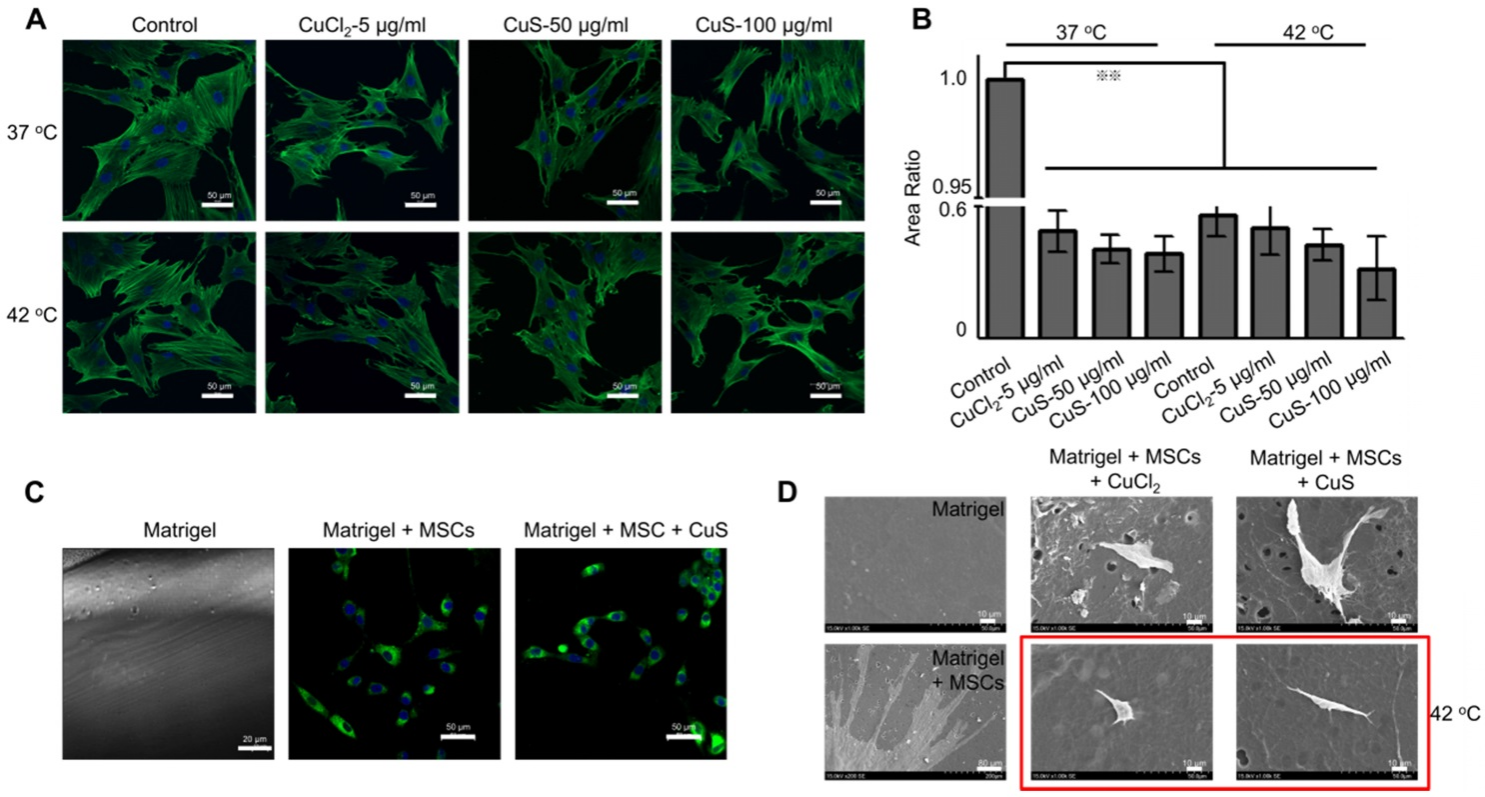
** The differentiation of MSCs treated with CuS@BSA.** Immunofluorescence images of β-actin in MSCs incubated in different concentrations of CuS@BSA for 48 h. (Blue: Nucleus; Green: β-actin. Scale bar = 50 μm). B) The area of MSCs were measured by ImageJ (**P < 0.01 by Student's t-test). C) Reflectance confocal image of Matrigel at 12-18 mg/ml (Scale bar =10 μm) and fluorescence images of MSCs infected with lentivirus carrying EGFP and Luciferase (Blue: Nucleus; Green: GFP. Scale bar =50 μm). D) SEM image of Matrigel and MSCs cultured in Matrigel with CuS@BSA and CuCl_2_, scale bar =10 μm.

Previous studies have revealed that Cu ions can induce the differentiation of MSCs and favor the regeneration of injured tissues. For a safer candidate, the potential of CuS@BSA nanoparticles inducing MSCs differentiation required further evaluation. We first observed the morphological change of MSCs induced by CuS@BSA via confocal microscopy by dual staining the actin and nucleus to identify the cells. For direct observation of the MSCs differentiation and morphology of each group, we performed an immunofluorescence method to mark β-actin and the nucleus (Figure 2A). The area of each cell was measured by Image J. The ratio of the MSCs area and nucleus decreased indicated that MSCs may be differentiating to fibroblasts (Figure 2B). The MSCs were then implanted into the Matrigel *in vitro* to stimulate actual growth condition. From the results of SEM and confocal microscope image, the morphology and area of MSCs changed and decreased in the group of CuS@BSA. Therefore, the MSCs can be grown in Matrigel and may also be induced to differentiate to fibroblasts by the introduction of CuS@BSA nanoparticles, which is similar to the results of MSCs cultured on the cell plate (Figure 2C and Figure 2D).

**Figure 3:**
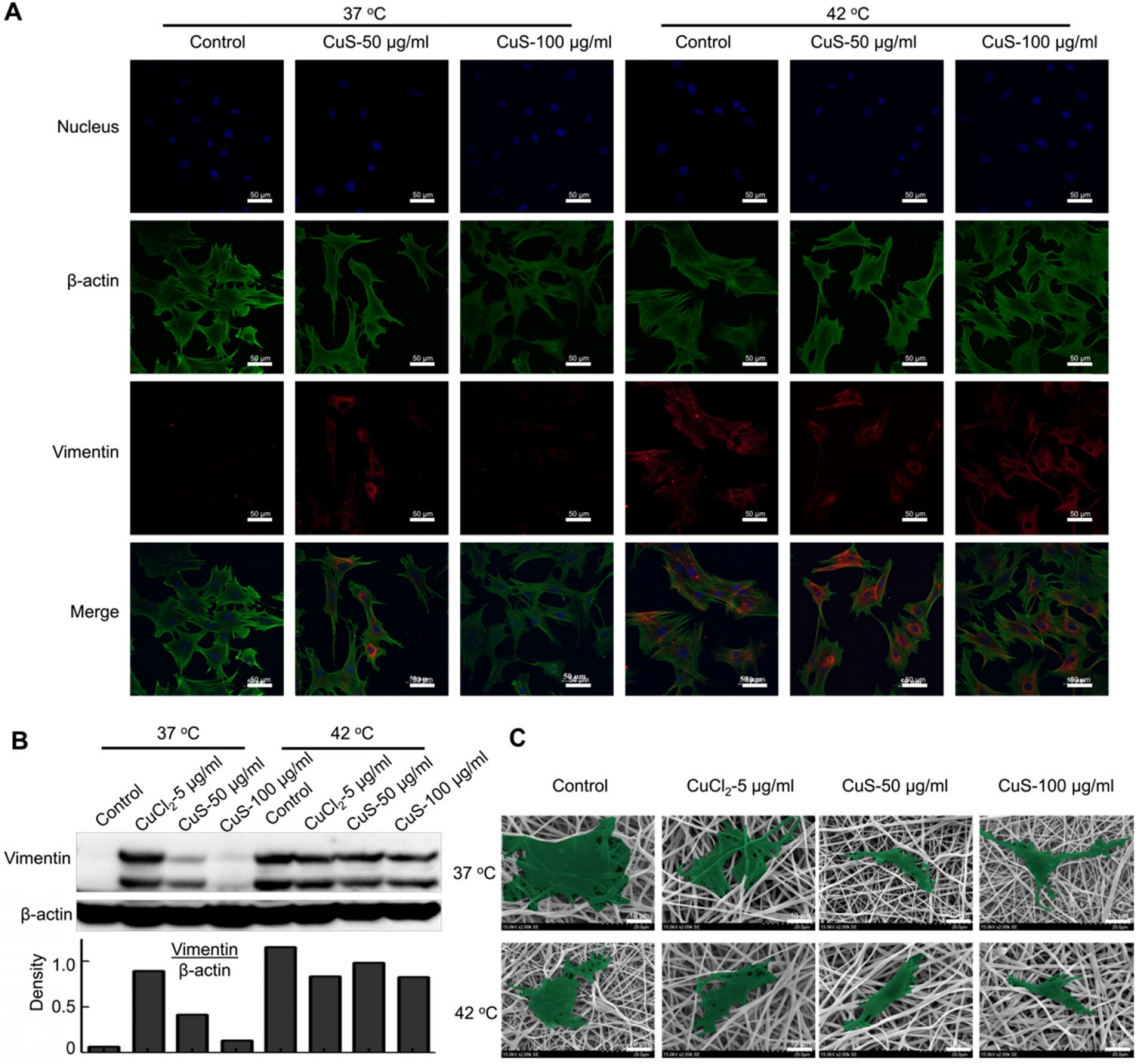
** The morphology and differentiation in MSCs treated with CuS@BSA.** A) The immunofluorescence images of β-actin and vimentin in MSCs incubated with each group for 48 h. (Blue: Nucleus; Green: β-actin; Red: Vimentin. Scale bar =50 μm). B) The expression of vimentin in MSCs by western blot detection. The intensity of vimentin/β-actin. C) SEM image of MSCs seeded in PLA electrospun film treated by CuCl_2_ and CuS@BSA for 3 days (20000×. Scale bar=10μm).

We further evaluated the expression of the vimentin, the marker of fibroblasts, in the differentiated MSCs by immunofluorescence detection (Figure 3A and Figure S1D). In addition, we used western boltting and qPCR assay to evaluate the protein and mRNA expression of vimentin (Figure 3B and Figure S1E). The introduction of Cu (both CuCl_2_ and CuS@BSA) stimulated the expression of vimentin in MSCs, but the expression of vimentin did not increase with the addition of Cu precursors. The cells treated with CuS@BSA at 100 μg/ml expressed less vimentin than other treatment groups. The expression of p-ERK/ERK was also detected (Figure S2A). These results are consistent with those of the immunofluorescence results.

Beyond the effects of CuS@BSA in MSCs differentiation, mild heating can also induce the differentiation of MSCs. In our previous report, we demonstrated that heating can upregulate the expression of some proteins of tumor cells, such as PD-L1, HSP^38^. Therefore, in MSCs differentiation, heating may also affect the expression of some proteins in MSCs and then induce the direction of MSC differentiation. Strikingly, while the culturing temperature was increased to 42 °C by water bath heating (controlled group) or by laser irradiation (CuS@BSA treated groups), high expression of vimentin was observed by confocal microscopy (Figure 3A). The semiquantitative results of the vimentin expression measured by western blotting assays also revealed similar results (Figure 3B and Figure S1E). For better observation, we cultured MSCs onto the electrospun PLA nanofiber membrane, and evaluated the effect of the introduction of heating on the morphological change of MSCs. Heating is an effective method to achieve MSCs differentiation to fibroblasts (Figure 3C). Both copper ions and temperature can induce MSCs differentiating to fibroblasts, but the high concentration of copper ion may cause an inhibiting effect for MSCs.

### CuS@BSA in prolonging the survival of MSCs *in vivo*

**Figure 4:**
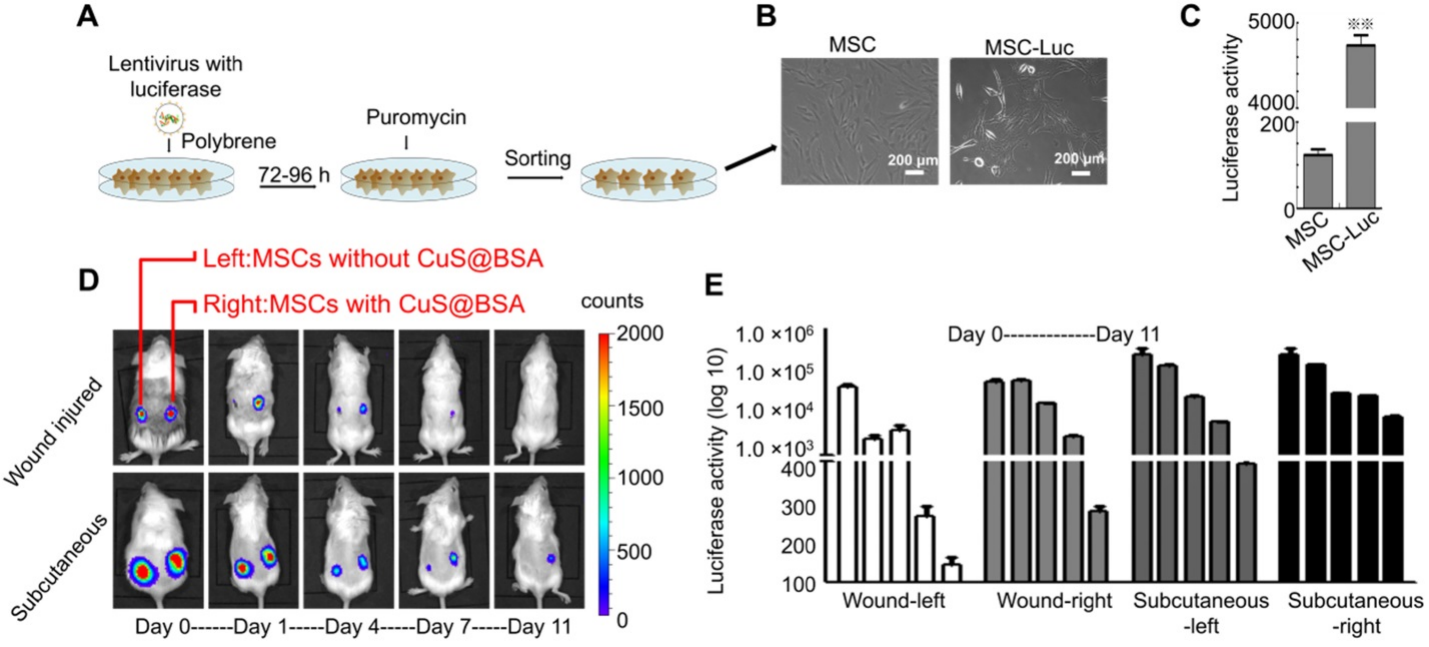
** The identification of MSC transfected with GFP and luciferase.** A) The scheme of the lentivirus infection process. B) The images of MSCs and MSCs transfected with lentivirus (Scale bar = 200 μm). C) The luciferase activity of MSCs and MSCs transfected with lentivirus detected by D-Luciferin sodium salt using a microplate reader (**P < 0.01 by Student's t-test). D) Bioluminescence images of BALB/c mice seeded with Matrigel and MSCs-luciferase at day 0,1,4,7,11 (Left: MSC without CuS@BSA, Right: MSC with CuS@BSA). E) The quantified fluorescence intensity of MSCs-luciferase *in vivo*.

The survival of MSCs *in vivo* is critical to improving the regeneration of injured tissues. Therefore, we further investigated the effect of CuS@BSA on the survival of MSCs *in vivo*. Before the evaluation of MSCs survival *in vivo*, we established MSCs with stably transfected luciferase by lentivirus infection to obtain MSCs-GFP-Luc for *in vivo* fluorescent imaging of implanted MSCs (Figure 4A), and the morphology of MSCs-GFP-Luc was no different than that of MSCs (Figure 4B). The activity of luciferase *in vitro* was also detected by D-Luciferin sodium salt (Figure 4C). MSCs-GFP-Luc and Matrigel mixture with/without CuS@BSA were seeded in BALB/c mice wound areas and subcutaneously, respectively. The activity of luciferase was detected at day 1, 4, 7, and 11 after cells were seeded in the wound area. The results revealed that the survival of MSCs with CuS@BSA was greater than MSCs without CuS@BSA (Figure 4D). The ratio of luciferase activity is shown in Figure 4E.

### *In vivo* Evaluation of Wound healing

**Figure 5:**
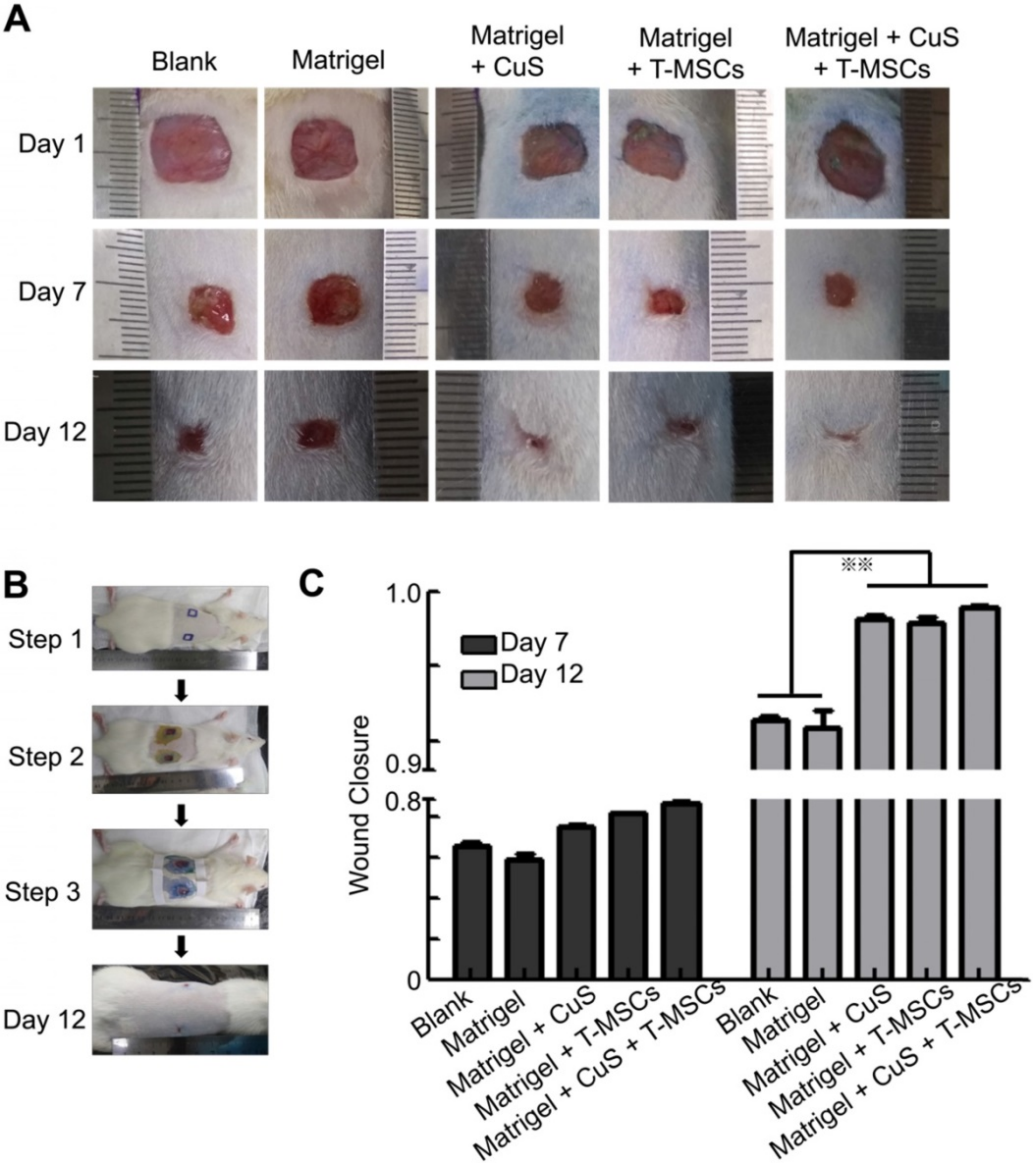
** The evaluation of CuS@BSA and preheated MSCs in wound healing.** A) Images of full-thickness skin defects in SD rat, saline, blank Matrigel, Matrigel + CuS, Matrigel + preheated MSCs, and Matrigel + CuS + preheated MSCs at 1, 7, and 14 days. B) The steps of the wound healing model. C) Wound closure percentages calculated by formula mentioned in methods and materials of different groups at 7 and 12 days (**P < 0.01 by Student's t-test).

**Figure 6:**
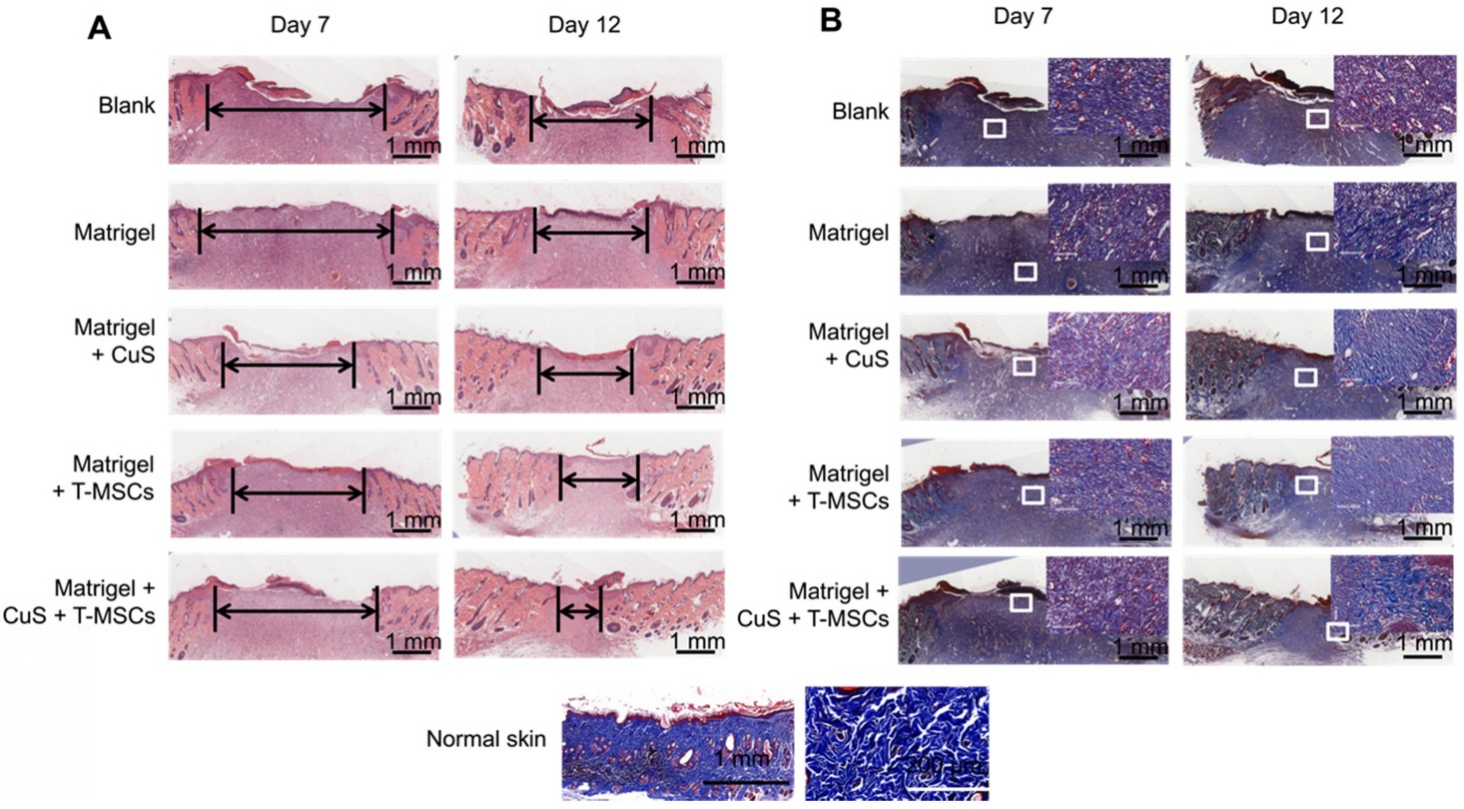
** Histologic evaluation of wound healing.** A,B) Images of H&E and Masson's trichrome staining of the different groups at 7 and 12 days (Scale bar = 1 mm). The black arrows indicate the degree of healing. The magnified view in Masson's trichrome staining showed the neonatal collagen (Scale bar=100 μm). The collagen arrangement in normal skin is shown for comparison.

The above results indicated that CuS@BSA combined with mild heating is a potential strategy to induce the differentiation of MSCs to fibroblasts, thus, improving the regeneration of injured skin tissues. Therefore, we first evaluated improving skin regeneration by the combination of preheated MSCs and CuS@BSA nanoparticles that were encapsulated in Matrigel. After establishing the skin wound model in SD rats, the rats were divided into several groups and treated with saline, blank Matrigel, Matrigel + CuS, Matrigel + preheated MSCs, and Matrigel + CuS+ preheated MSCs. Images of the wound healing were captured at day 1, 7 and 12, and the wound closure of each mouse calculated using Image J. Based on the results, the wound of the group treated with Matrigel + CuS + preheated MSCs closed significantly faster than the other groups (Figure 5). The H&E and Masson's trichrome staining results of the wounds are shown in Figure 6.

The degree of healing was marked by the arrow under the full-thickness section with derma. The new endothelium treated with Matrigel + CuS + preheated MSCs was significantly faster than the defects treated with MSCs or CuS only at both day 7 and 12. Masson's trichrome staining also indicated the collagen differences in the defect repair. CuS@BSA and MSCs both stimulated extensive collagen deposition, and much thicker wavy collagen fibers were observed in the wounds. The newly formed collagen deposition in the group treated with Matrigel + CuS + preheated MSCs were more similar with the normal skin (Figure 6). The results demonstrated that the combination of CuS@BSA and preheating of MSCs is a potential strategy for skin wound regeneration.

Besides, we compared the difference between preheated MSCs and MSCs with CuS@BSA or CuCl_2_ for wound healing. We found that the wound of the group treated with preheated MSCs closed significantly faster than the groups with same addition but no preheated MSCs (Figure S3). It did reveal that mild temperature promote the proliferation of MSCs.

**Figure 7:**
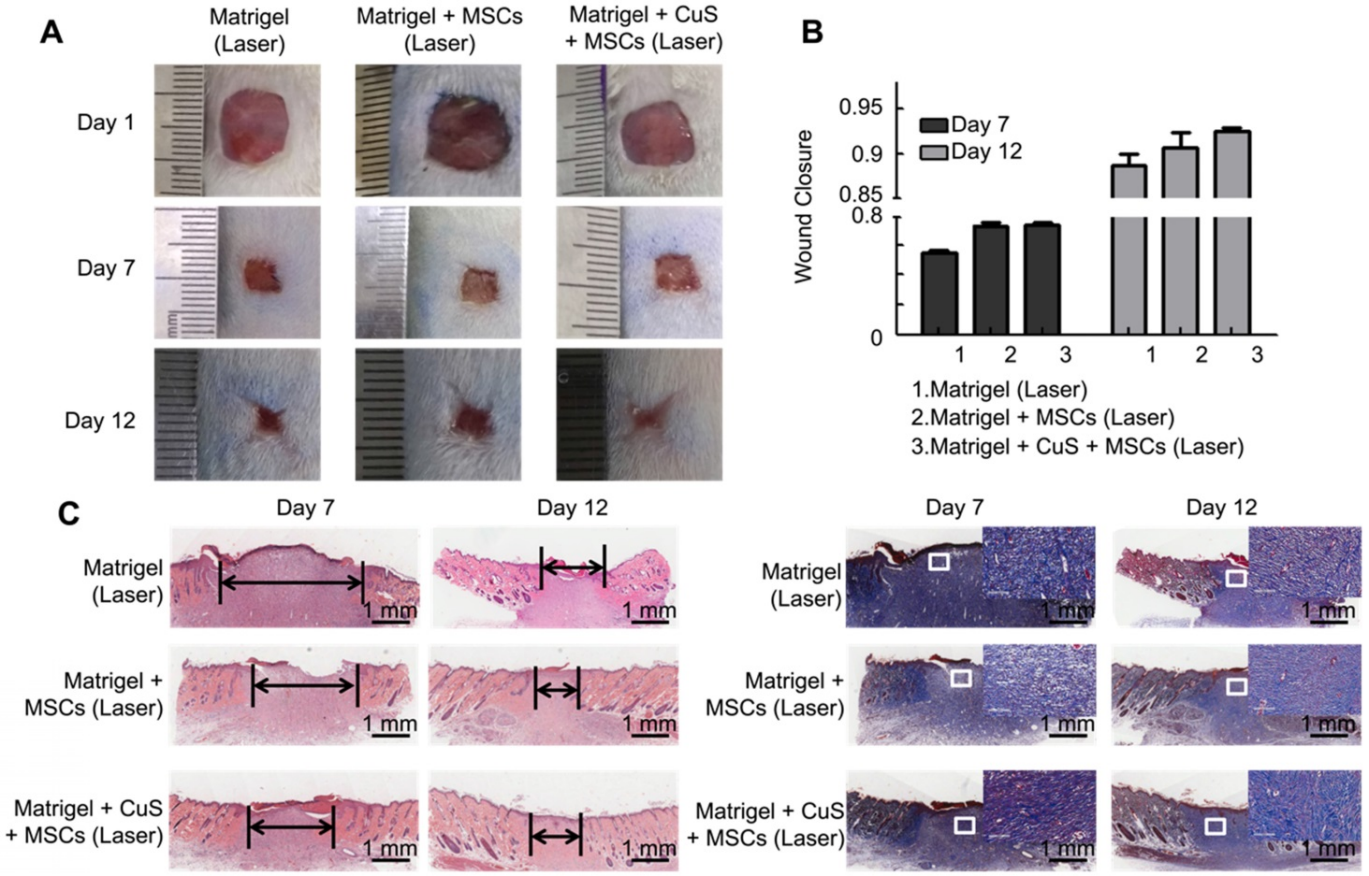
** The evaluation of CuS@BSA under a laser in wound healing.** A) Images of full-thickness skin defects in SD rat, blank Matrigel with laser, Matrigel + MSCs with laser, and Matrigel + CuS@BSA + MSCs with laser at 1, 7, and 14 days. B) Wound closure percentages calculated by formula mentioned in methods and materials of different groups at 7 and 12 days. C) Images of H&E and Masson's trichrome staining of the different groups at 7 and 12 days (Scale bar = 1 mm). The black arrows indicate the degree of healing. The magnified view in Masson's trichrome staining showed neonatal collagen (Scale bar=100 μm).

Beyond preheating, we further evaluated the influence of *in situ* mild heating of MSCs by laser irradiation to the regeneration of skin wound. After the MSCs were collected, they were mixed with CuS@BSA containing Matrigel, and placed onto the wound site. While the mixture was in the gel state, the gel was irradiated by a 980 nm laser (0.8 W/cm^2^). The MSCs that did not undergo preheating but NIR irradiating exhibited weaker improvement in the skin regeneration, although it still favored the closure of the wound while comparing with the saline-treated group (Figure 7). The results illustrate *in situ* mild heating also improved the regeneration of the skin wound by stimulating the generation and formation of collagen deposition in the wound site. It further demonstrated that the CuS@BSA nanoparticles self-synergistically improved the regeneration of injured skin by combining with CuS@BSA mediated photothermal generation and their potential in inducing MSCs differentiation to fibroblasts. However, the input laser power should be strictly controlled. While the input power of the laser was further increased, *in situ* heating seems to impede the closure of the injured wound (Figure S4). Overheating of Matrigel may cause damage to the encapsulated MSCs and surrounding Matrigel-tissues interfaces.

### Toxicity of CuS@BSA *in vivo*

**Figure 8:**
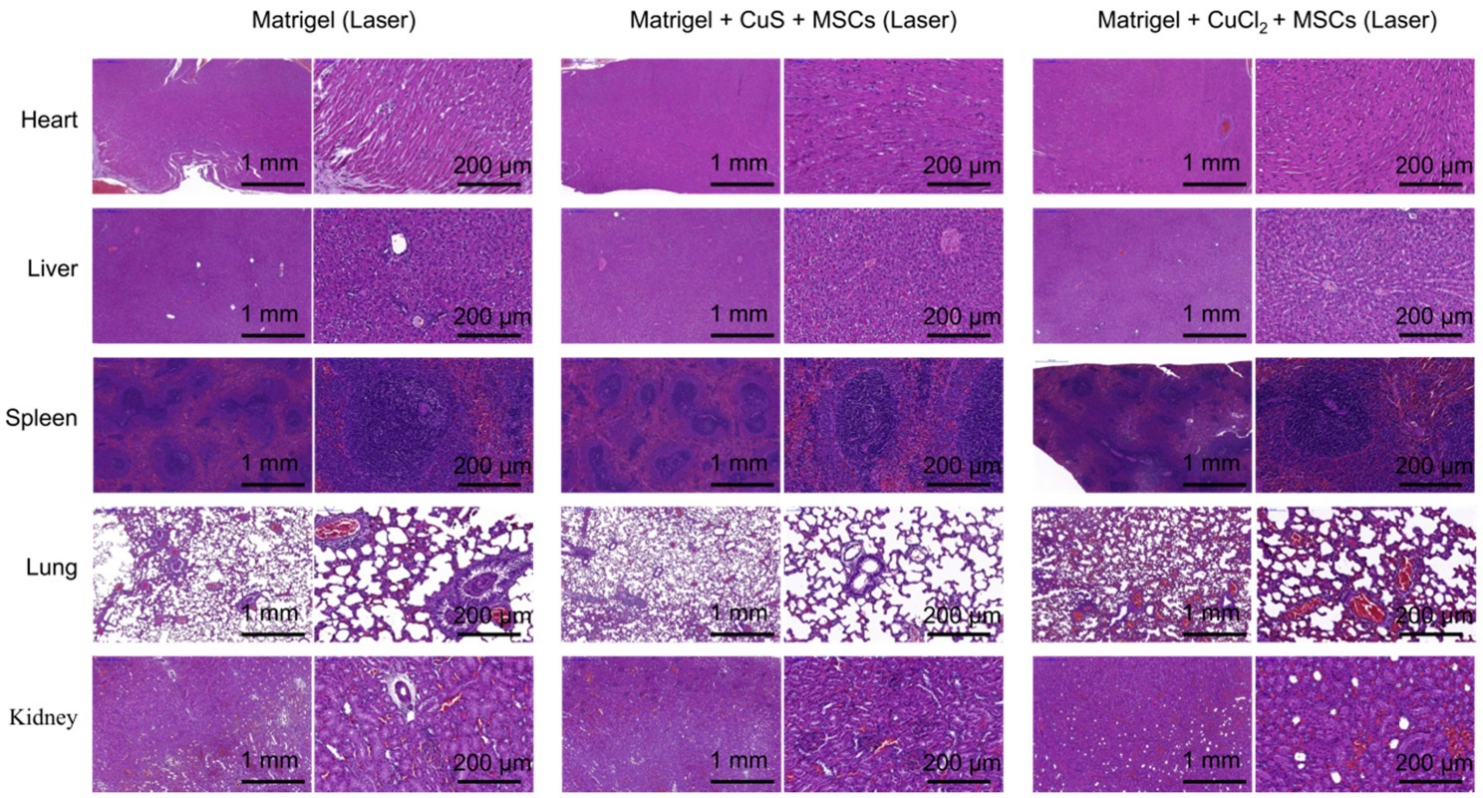
H&E histologic evaluation of major organs (heart, liver, spleen, lung, and kidney) of the Matrigel, Matrigel + CuS@BSA + MSCs with laser, and Matrigel CuCl_2_ + MSCs with laser groups.

We further examined the toxicity of CuS@BSA to the major organs of the mice *in vivo*. By pathological examination of the major organs, we found no obvious toxicity in the heart, liver, spleen, lung, or kidney of mice treated with CuS@BSA, while the mice treated with CuCl_2_ did exhibit some kidney damage and inflammation in the lung tissue (Figure 8). Therefore, the CuS@BSA nanoparticles are a promising substitution of Cu ions.

## Conclusions

In summary, the CuS@BSA nanoparticles exhibited similar potential in inducing MSCs differentiation to fibroblasts as Cu ions. CuS@BSA nanoparticles can serve as a suitable agent to control the direction of MSC differentiation. Furthermore, the introduction of heating may favor the differentiation of MSCs to fibroblasts. Based on the *in vitro* results, either the CuS@BSA nanoparticles themselves or the heating stimulated the expression of vimentin in MSCs, which further directed the MSCs to fibroblasts. By establishing an injured wound model *in vivo*, we further revealed that the combination of CuS@BSA nanoparticles and thermal treatment synergistically improved the closure of a wound. Therefore, MSCs combined with CuS@BSA are a promising wound dressing in the reconstruction of a full-thickness skin injury.

## Supplementary Material

Supplementary figures.

## Figures and Tables

**Scheme 1:**
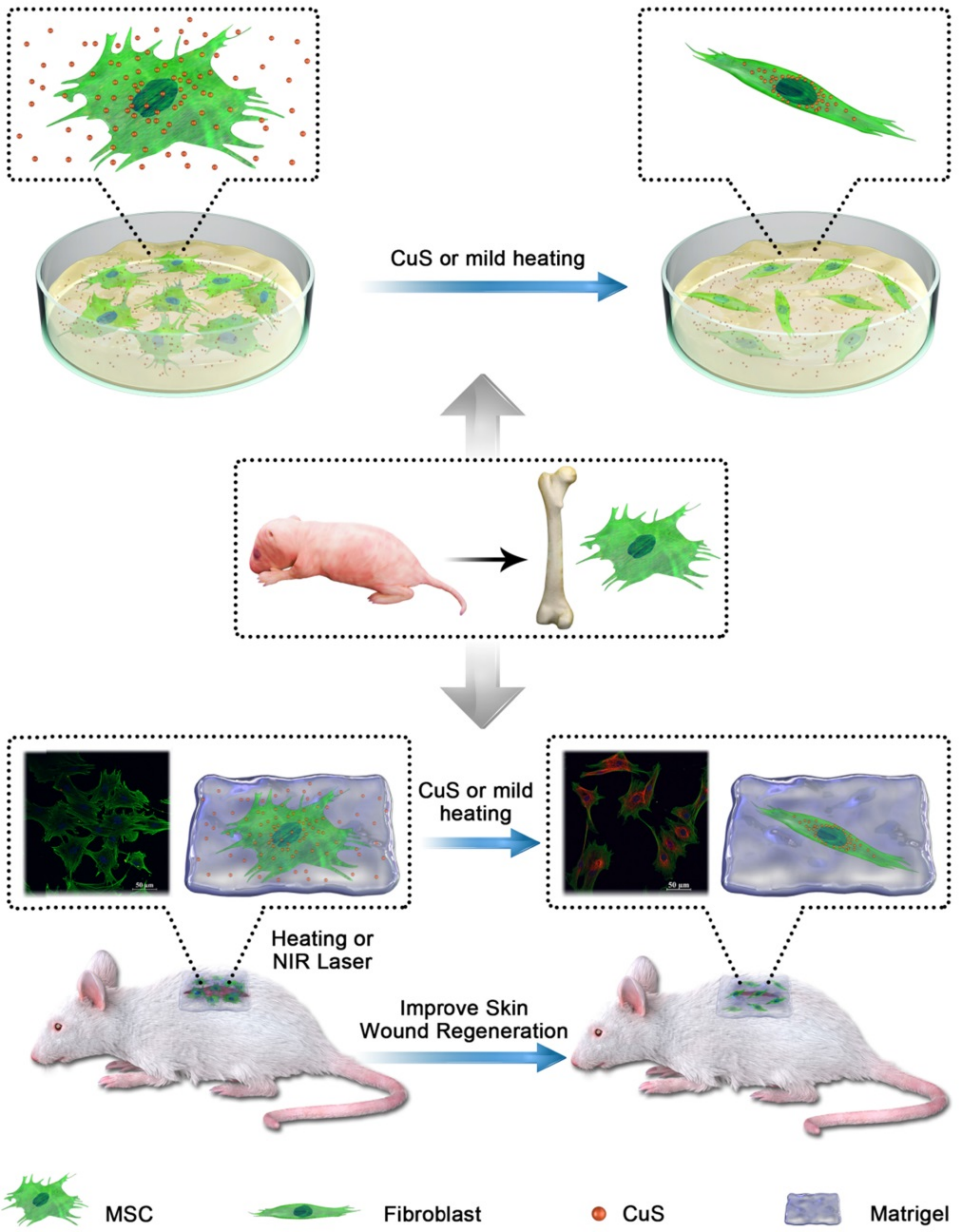
Schematic illustration of Mesenchymal stem cells (MSCs) isolated from newborn SD/BALB/c mice bone marrow were treated with CuS@BSA or mild heating, which can improve the wound clourse though promote the MSCs proliferation and differentiation to fibroblasts.

## Abbreviations

MSCs: mesenchymal stem cells
BM-MSCs: bone marrow-derived mesenchymal stromal cells
SDR: scaffold for dermal regeneration
ECM: extracellular matrix
VEGF: vascular endothelial growth factor
Ctrl 1: copper transporter receptor1
ERK: extracellular regulated protein kinases
MEK: MAP kinase/ ERK kinase
HIF-1α: hypoxia-inducible factor
PTAs: photothermal agents
NIR: near infrared
CSO HMS: copper silicate hollow microsphere
PTT: photothermal therapy
HRP: horseradish peroxidase
DLS: dynamic light scattering
TEM: transmission electron microscope
DMEM: Dulbecco's modified Eagle medium
FBS: fetal bovine serum
MNC: mononuclear cell
GFP: green fluorescent protein
PLA: polylactic acid
SEM: scanning electronic microscopy
