# Supplementary material for: Ultrasmall CuS@BSA nanoparticles with mild photothermal conversion synergistically induce MSCs-differentiated fibroblast and improve skin regeneration
Source: Theranostics. 2020 Jan 1;10(4):1500–13. doi: 10.7150/thno.39471 (PMC6993219; doi:10.7150/thno.39471)
Supplement: Supplementary file 1 — Supplementary figures. [file thnov10p1500s1.pdf]

Supporting Information

Ultrasmall CuS @BSA nanoparticles together with mild photothermal conversion synergistically induce MSCs-differentiated fibroblast and improve skin regeneration

Yao Xiao [1], Jinrong Peng [1], Qingya Liu [1], Lijuan Chen [2], Kun Shi[1], Ruxia Han[1], Qian Yang[3], Lin Zhong[1], Ruoyu Zha[1], Ying Qu[1], Zhiyong Qian [1,*]

[1] State Key Laboratory of Biotherapy and Cancer Center, West China Hospital, West China Medical School, Sichuan University and Collaborative Innovation Center of Biotherapy, Chengdu 610041, China

[2] The Department of Radiology, Henan Key Laboratory of Neurological Imaging, Henan Provincial People's Hospital & the People's Hospital of Zhengzhou University, Zhengzhou 450003, China

[3] The School of Pharmacy, Chengdu Medical College, Chengdu, 610500, China Corresponding author: State Key Laboratory of Biotherapy and Cancer Center, West China Hospital, West China Medical School, Sichuan University and Collaborative Innovation Center, Chengdu 610041, China

Email address: anderson-qian@163.com or zhiyongqian@scu.edu.cn (Z. Qian)

Figure S1

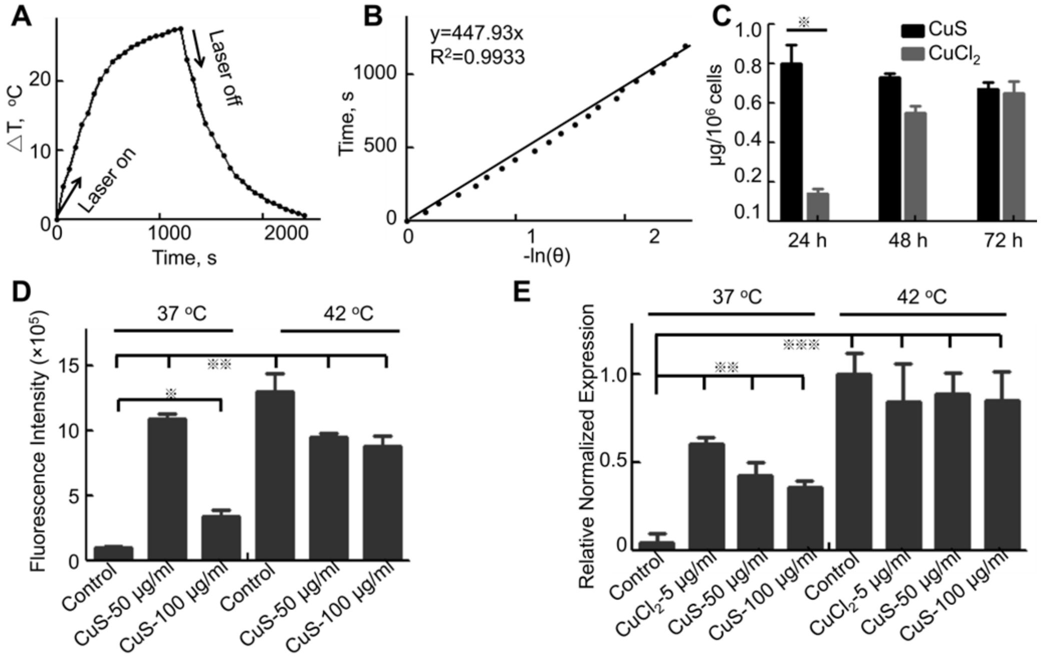

Figure S1. A) The temporal temperature variation CuS@BSA solution. The solution is irradiated for 20 min using a 1.42 W/cm^2 and cooled to room temperature under ambient environment. B) Time constant for heat transfer from the system determined by the linear time data from the temperature of the heating-cooling period. C) The uptake of CuS@BSA and $CuCl_2$ for MSCs at 24h, 48h, 72h, measured by ICP-MS (*P < 0.05 by Student's t-test). D) The fluorescence intensity of vimentin was quantitatively by image J (*P < 0.05 and **P < 0.01 by Student's t-test). E) The expression of vimentin mRNA was measured by qPCR. (**P < 0.01 and ***P < 0.001 by Student's t-test).

Figure S2

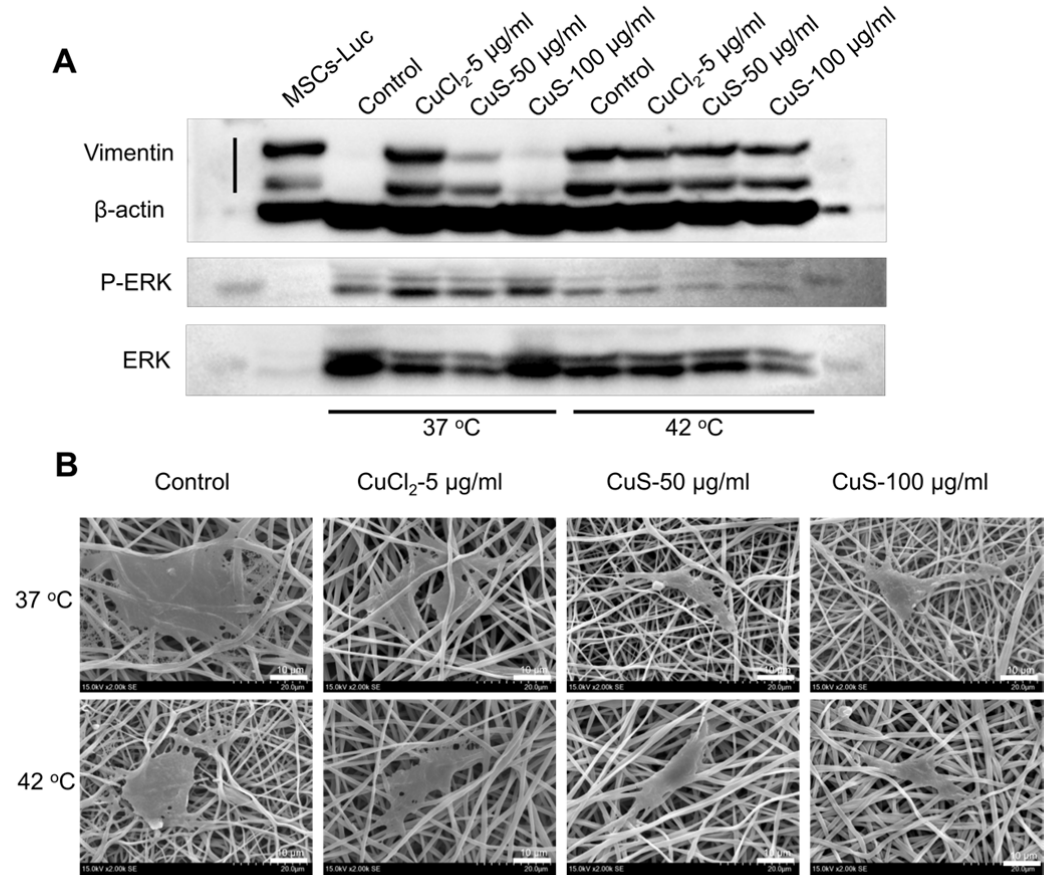

Figure S2. A) The expression of vimentin and ERK in MSCs-Luc and MSCs treated with CuS@BSA and $CuCl_2$. B) The SEM image of MSCs seeded in PLA electrospun film treated by $CuCl_2$ and CuS@BSA for 3 days (20000×. Scale bar= 10 μm).

Figure S3

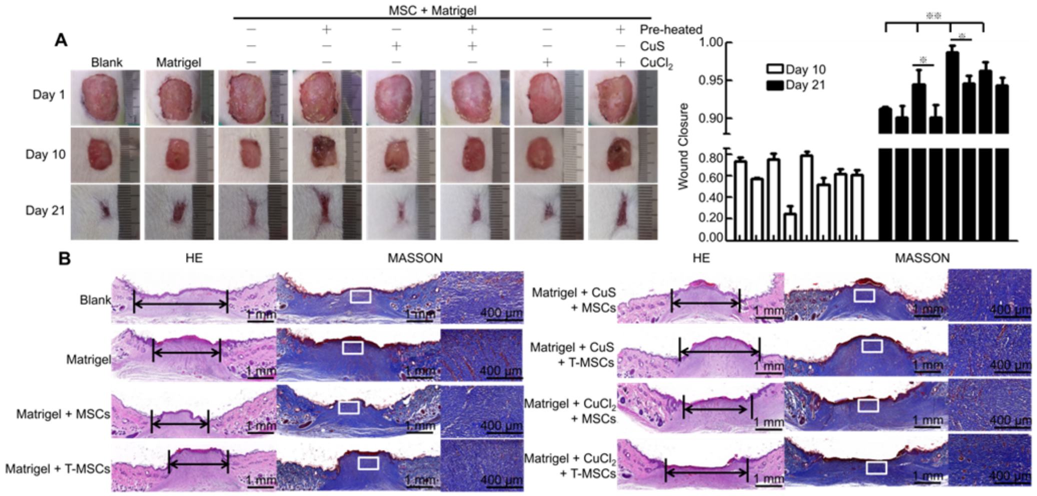

Figure S3. A) Images of full-thickness skin defects in SD rat, saline, Matrigel, Matrigel+ MSCs, Matrigle+ preheated MSCs, Matrigel+ MSCs+ CuS@BSA, Matrigel+ preheated MSCs+ CuS@BSA, Matrigel+ MSCs+ $CuCl_2$, Matrigel+ preheated MSCs+ $CuCl_2$ at 1, 10 and 21 days. Wound closure percentages were calculated by the formula mentioned in the methods and materials of different groups at day 10 and 21(*$P < 0.05$ and **$P < 0.01$ by Student's t-test). B) An image of H&E and Masson's trichrome staining of the different groups at 21 days.

Figure S4

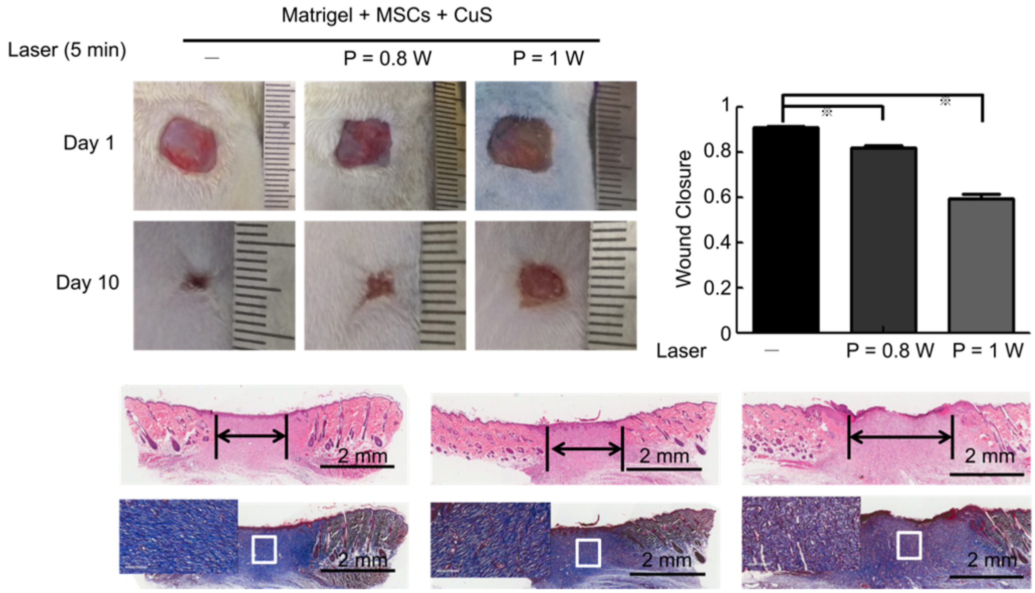

Figure S4. The evaluation of NIR for wound healing. A) Images of full-thickness skin defects in SD rat, Matrigel with CuS@BSA and MSCs, Matrigel with CuS@BSA and MSCs after NIR at power 0.8 W, Matrigel with CuS@BSA and MSCs after NIR at power 1 W at Day 1 and Day 10. Wound closure percentages were calculated by the formula mentioned in the methods and materials of different groups at day 10. B) An image of H&E and Masson's trichrome staining of the different groups at 10 days(*$P < 0.05$ by Student's t-test).
